# Predictive value of metabolic and inflammatory indices (TGI, TG/HDL-C, and PIV) for complications in type 2 diabetes mellitus: A retrospective cohort study

**DOI:** 10.1097/MD.0000000000047725

**Published:** 2026-02-20

**Authors:** Yahya Urkmez, Mehmet Karacali, Esra Urkmez Kilinc

**Affiliations:** a Department of Internal Medicine, Gaziantep City Hospital, Gaziantep, Turkey; b Department of Internal Medicine, School of Medicine, Gaziantep University, Gaziantep, Turkey; c Department of Pediatrics, Cengiz Gökçek Gynecology and Pediatrics Hospital, Gaziantep, Turkey.

**Keywords:** diabetic complications, pan-immune-inflammation value, risk prediction, TG/HDL-C ratio, triglyceride-glucose index, type 2 diabetes mellitus

## Abstract

This study aimed to investigate the association between the triglyceride-to-HDL cholesterol ratio (TG/HDL-C), triglyceride-glucose index (TGI), and pan-immune-inflammation value (PIV) with the presence of complications in patients with type 2 diabetes mellitus (T2DM). This retrospective cohort study included 450 patients diagnosed with T2DM between October 2023 and April 2025 at the Department of Internal Medicine, Gaziantep City Training and Research Hospital. Demographic, clinical, and laboratory parameters were collected. TG/HDL-C, TGI, and PIV were calculated using standard formulas. Patients were divided into groups based on the presence or absence of diabetic complications. Comparative, correlation, logistic regression, and ROC analyses were performed to assess the predictive value of these indices. Diabetic complications were present in 63.3% of patients. Patients with complications had significantly higher values of TG/HDL-C (5.24 ± 1.57 vs 4.31 ± 1.21, *P* < .001), TGI (9.16 ± 0.53 vs 8.68 ± 0.49, *P* < .001), and PIV (471.2 vs 351.4, *P* < .001). TGI (*R* = 0.68), PIV (*R* = 0.65), and TG/HDL-C (*R* = 0.62) showed moderate to strong statistically significant correlations with the presence of diabetic complications. In logistic regression, TGI (OR: 2.12), PIV (OR: 1.94), and TG/HDL-C (OR: 1.83) remained independent predictors (all *P* < .001). ROC analysis revealed high diagnostic accuracy for TGI (AUC = 0.813), PIV (AUC = 0.794), and TG/HDL-C (AUC = 0.782). TGI, TG/HDL-C ratio, and PIV are significantly associated with the presence of diabetic complications and demonstrate strong individual predictive value. Moreover, a combined model integrating all 3 indices further improved diagnostic accuracy (AUC = 0.846), supporting their potential use as complementary tools in comprehensive risk stratification for patients with type 2 diabetes mellitus.

## 1. Introduction

Type 2 diabetes mellitus (T2DM) is a complex and progressive metabolic disorder characterized by insulin resistance, impaired insulin secretion, and chronic low-grade inflammation. It has become a major global health challenge, affecting over 500 million individuals worldwide and contributing substantially to morbidity, mortality, and healthcare costs.^[1,2]^ While hyperglycemia is a hallmark of T2DM, emerging evidence suggests that chronic inflammation and metabolic dysregulation play crucial roles in the pathogenesis of diabetic complications.^[3–7]^

Given the high burden of diabetes-related complications, there is a growing need for reliable and easily accessible biomarkers that can support early risk stratification. Traditional indicators such as fasting glucose or HbA1c offer useful information about glycemic status but may not adequately capture the underlying metabolic and inflammatory disturbances that contribute to complications. Recently, several indices derived from routine laboratory tests have gained attention as practical tools for evaluating insulin resistance, dyslipidemia, and systemic inflammation.^[6,8]^

In this context, several novel indices derived from routine blood parameters have gained attention. The triglyceride-to-HDL cholesterol (TG/HDL-C) ratio is a widely studied surrogate marker for insulin resistance and atherogenic dyslipidemia. Elevated TG/HDL-C ratios have been associated with increased cardiovascular risk and metabolic syndrome, particularly in individuals with T2DM.^[9–12]^ Similarly, the triglyceride-glucose index (TGI), calculated using fasting triglyceride and glucose levels, has been validated as a reliable and practical indicator of insulin resistance, comparable to HOMA-IR in various populations.^[13–15]^

In parallel, systemic inflammation has been increasingly recognized as a key driver of diabetes-related complications. The pan-immune-inflammation value (PIV), which integrates neutrophil, monocyte, platelet, and lymphocyte counts, reflects a comprehensive immune-inflammatory status and has been recently proposed as a prognostic marker in oncology and cardiovascular research.^[10,16]^ However, its role in the context of metabolic diseases, particularly T2DM, remains underexplored.

Although several studies have examined TG/HDL-C, TGI, or PIV independently, few investigations have explored their combined relevance within the same clinical population. Because metabolic dysfunction and chronic low-grade inflammation act synergistically in the development of diabetic complications, incorporating both metabolic and inflammatory indices may offer superior risk stratification compared to evaluating each marker type in isolation. To our knowledge, no previous study has simultaneously evaluated TG/HDL-C, TGI, and PIV in the prediction of diabetes-related complications, and the present study aims to address this gap by assessing their comparative and complementary predictive value in patients with T2DM.

The aim of this study was to investigate the association of TG/HDL-C ratio, TGI, and PIV with clinical status, laboratory parameters, and diabetes-related complications in patients with type 2 diabetes. By leveraging easily accessible laboratory data, we sought to identify valuable markers for enhancing clinical risk assessment and individualized disease management in this high-risk population.

## 2. Methods

### 2.1. Study design and population

This retrospective cohort study was conducted in the Department of Internal Medicine at Gaziantep City Training and Research Hospital. The study included adult patients diagnosed with T2DM who presented between October 1, 2023, and April 18, 2025. The diagnosis of T2DM was based on the criteria established by the American Diabetes Association (ADA).^[17]^

Patients were eligible for inclusion if they were aged 18 years or older, had been followed for at least 1 year with a diagnosis of T2DM, and had complete clinical and laboratory data available. Patients with type 1 or gestational diabetes, acute infections, malignancies, autoimmune or chronic inflammatory diseases, pregnancy, lactation, or incomplete data were excluded from the study.

### 2.2. Data collection

Demographic characteristics (age, sex, body mass index (BMI), and duration of diabetes), comorbidities, and laboratory values were retrieved retrospectively from electronic medical records. Laboratory parameters included fasting plasma glucose, triglycerides, high-density lipoprotein cholesterol (HDL-C), neutrophil, lymphocyte, monocyte, and platelet counts. Three composite indices were calculated: the triglyceride-to-HDL-C ratio (TG/HDL-C) was computed by dividing TG by HDL-C levels; the triglyceride-glucose (TG) index was derived using the formula ln [TG (mg/dL) × fasting glucose (mg/dL)/2], and the pan-immune-inflammation value (PIV) was calculated by multiplying neutrophil, monocyte, and platelet counts and dividing the product by the lymphocyte count. In this calculation, neutrophils, lymphocytes, and monocytes were measured in 10^3^/μL, and platelets were measured in 10^3^/μL, consistent with standard automated hematology analyzer outputs.

A formal a priori sample size calculation was not performed because the study included all consecutive eligible patients within the specified study period, yielding a final sample size of 450 individuals. To evaluate whether this sample size was sufficient, a post hoc power assessment was conducted based on the effect sizes observed in the logistic regression models. Using the odds ratio (OR) of the primary predictor (TGI; OR = 2.1), the achieved power exceeded 90% at an alpha level of 0.05, indicating that the study was adequately powered to detect clinically meaningful associations. Therefore, the sample size was considered sufficient for the statistical analyses performed.

### 2.3. Statistical analysis

Statistical analyses were performed using IBM SPSS Statistics (version 27). The Shapiro–Wilk test was used to assess the normality of continuous variables. Data were expressed as mean ± standard deviation for normally distributed variables and as median (interquartile range) for non-normally distributed variables. Frequencies and percentages were used for categorical variables. Between-group comparisons were conducted using the independent samples t-test or Mann–Whitney *U* test for continuous variables, and the chi-square or Fisher exact test for categorical variables. Correlations between TG/HDL-C, TGI, PIV, clinical/laboratory parameters, and diabetic complications were assessed using Pearson or Spearman correlation coefficients. Logistic regression analyses were performed to identify independent predictors of diabetic complications. For multivariable logistic regression, all clinically relevant variables – including age, sex, BMI, HbA1c, diabetes duration, and the 3 primary indices (TGI, TG/HDL-C, and PIV) – were initially entered into the model. A backward stepwise selection procedure (Wald method) was applied, removing variables with *P* > .10 to minimize overfitting. The final model retained HbA1c, diabetes duration, TGI, TG/HDL-C, and PIV, which are presented in Table 4.

To minimize residual confounding, key clinical variables known to influence diabetic complications (age, sex, BMI, HbA1c, diabetes duration) were included in the multivariable models. Medication use and comorbidities were reviewed in electronic records; although not incorporated as individual covariates due to inconsistent documentation and potential overfitting, no systematic differences were observed between groups. Lifestyle-related variables (diet, smoking intensity, and physical activity) were not consistently available, representing an inherent limitation of the retrospective design.

Receiver operating characteristic (ROC) curves were generated to evaluate the predictive performance of each index, and the area under the curve (AUC) was calculated. Additionally, a combined predictive model incorporating TGI, TG/HDL-C, and PIV was constructed using multivariable logistic regression. Predicted probabilities from this model were used to generate a combined-model ROC curve. The AUC of the combined model was compared with that of each individual index to determine whether integrating metabolic and inflammatory markers improved discriminatory performance.

## 3. Results

Demographic, clinical, and laboratory characteristics of the study population were shown in Table 1. A total of 450 patients with T2DM were included in the study, with a mean age of 58.4 ± 10.2 years. Of the participants, 54.2% were female (n = 244) and 45.8% were male (n = 206). The mean duration of diabetes was 7.3 ± 4.8 years, and the mean BMI was 29.1 ± 3.5 kg/m^2^. The average HbA1c level was 8.1 ± 1.2%. Regarding metabolic parameters, the mean fasting glucose level was 147.6 ± 38.2 mg/dL, the mean triglyceride level was 196.5 ± 52.3 mg/dL, and the mean HDL-C level was 41.8 ± 10.7 mg/dL. The mean TG/HDL-C ratio was calculated as 4.89 ± 1.53, and the mean TGI was 8.94 ± 0.54. Inflammatory markers included a median PIV value of 412.6 (interquartile range: 320.3–558.7), with neutrophil, lymphocyte, monocyte, and platelet counts averaging 5.2 ± 1.8 × 10^3^/μL, 2.1 ± 0.6 × 10^3^/μL, 0.54 ± 0.18 × 10^3^/μL, and 265 ± 75 × 10^3^/μL, respectively.

**Table 1:** Demographic, clinical, and laboratory characteristics of the study population.

Parameter	Patients (N = 450) Mean ± SD, n (%)
Age (yr)	58.4 ± 10.2
Gender	
Female	244 (54.2%)
Male	206 (45.8%)
Diabetes duration (yr)	7.3 ± 4.8
Body mass index (kg/m^2^)	29.1 ± 3.5
HbA1c (%)	8.1 ± 1.2
Fasting glucose (mg/dL)	147.6 ± 38.2
Triglycerides (mg/dL)	196.5 ± 52.3
HDL-C (mg/dL)	41.8 ± 10.7
TG/HDL-C ratio	4.89 ± 1.53
TGI	8.94 ± 0.54
PIV (median, IQR)	412.6 (IQR: 320.3–558.7)
Neutrophils (10^3^/μL)	5.2 ± 1.8
Lymphocytes (10^3^/μL)	2.1 ± 0.6
Monocytes (10^3^/μL)	0.54 ± 0.18
Platelets (10^3^/μL)	265 ± 75
Complications	285 (63.3%)
Diabetic nephropathy	68 (15.1%)
Diabetic retinopathy	72 (16.0%)
Peripheral neuropathy	85 (18.9%)
Macrovascular complications	60 (13.3%)

HbA1c = glycated hemoglobin; TG/HDL-C = triglyceride-to-HDL cholesterol, TGI = triglyceride-glucose index, PIV = pan-immune-inflammation value; IQR = interquartile range.

Of the total population, 285 patients (63.3%) presented with one or more diabetic complications. The most frequent complication was peripheral neuropathy (18.9%), followed by diabetic retinopathy (16.0%), diabetic nephropathy (15.1%), and macrovascular complications such as coronary artery disease and stroke (13.3%) (Table 1).

Comparison of parameters according to the presence of complications were shown in Table 2. Among the study population, 285 patients (63.3%) had at least 1 diabetic complication. When comparing patients with complications (n = 285) to those without (n = 165), several significant differences were observed. Patients with complications were older (61.2 ± 10.4 vs 56.1 ± 9.8 years, *P* < .001) and had a longer duration of diabetes (8.5 ± 5.1 vs 5.9 ± 3.6 years, *P* < .001). The complication group also showed significantly higher HbA1c levels (8.5 ± 1.3 vs 7.6 ± 0.9%, *P* < .001) and BMI values (30.0 ± 3.7 vs 28.4 ± 3.2 kg/m^2^, *P* = .006). Metabolic parameters such as fasting glucose (162.1 ± 39.5 vs 134.2 ± 33.6 mg/dL, *P* < .001), triglycerides (207.4 ± 53.6 vs 183.2 ± 47.1 mg/dL, *P* < .001), and TG/HDL-C ratio (5.24 ± 1.57 vs 4.31 ± 1.21, *P* < .001) were significantly elevated in the complication group, whereas HDL-C levels were lower (39.2 ± 10.4 vs 45.3 ± 9.8 mg/dL, *P* = .002). Similarly, the TGI was significantly higher in patients with complications (9.16 ± 0.53 vs 8.68 ± 0.49, *P* < .001). Regarding inflammatory markers, the PIV value was markedly higher in the complication group (471.2 vs 351.4, *P* < .001), accompanied by increased neutrophil and monocyte counts (5.6 ± 1.9 vs 4.7 ± 1.6, *P* < .001; 0.59 ± 0.19 vs 0.48 ± 0.15, *P* = .004, respectively) and decreased lymphocyte counts (1.8 ± 0.5 vs 2.4 ± 0.7, *P* < .001). Platelet counts were also significantly higher in the complication group (273 ± 78 vs 258 ± 70, *P* = .021) (Table 2.).

**Table 2:** Comparison of parameters according to the presence of complications.

Parameter	Complications		*P* value
	No (N = 165) Mean ± SD, n (%)	Yes (N = 285) Mean ± SD, n (%)	
Age (yr)	56.1 ± 9.8	61.2 ± 10.4	<.001
Gender – female	104 (63.0%)	140 (49.1%)	.018
Gender – male	61 (37.0%)	145 (50.9%)	.018
Diabetes duration (yr)	5.9 ± 3.6	8.5 ± 5.1	<.001
Body mass index (kg/m^2^)	28.4 ± 3.2	30.0 ± 3.7	.006
HbA1c (%)	7.6 ± 0.9	8.5 ± 1.3	<.001
Fasting glucose (mg/dL)	134.2 ± 33.6	162.1 ± 39.5	<.001
Triglycerides (mg/dL)	183.2 ± 47.1	207.4 ± 53.6	<.001
HDL-C (mg/dL)	45.3 ± 9.8	39.2 ± 10.4	.002
TG/HDL-C Ratio	4.31 ± 1.21	5.24 ± 1.57	<.001
TGI	8.68 ± 0.49	9.16 ± 0.53	<.001
PIV (median, IQR)	351.4 (280.2–460.6)	471.2 (390.5–622.1)	<.001
Neutrophils (10^3^/µL)	4.7 ± 1.6	5.6 ± 1.9	<.001
Lymphocytes (10^3^/µL)	2.4 ± 0.7	1.8 ± 0.5	<.001
Monocytes (10^3^/µL)	0.48 ± 0.15	0.59 ± 0.19	.004
Platelets (10^3^/µL)	258 ± 70	273 ± 78	.021

HbA1c = glycated hemoglobin; TG/HDL-C = triglyceride-to-HDL cholesterol, TGI = triglyceride-glucose index, PIV = pan-immune-inflammation value; IQR = interquartile range.

Correlation analysis between clinical and laboratory parameters and the presence of diabetic complications were shown in Table 3. The most statistically significant positive correlations were observed for diabetes duration (*R* = 0.43), HbA1c (*R* = 0.36), TG/HDL-C (*R* = 0.62), TGI (*R* = 0.68), and PIV (*R* = 0.65). While TG/HDL-C, TGI, and PIV demonstrated moderate to strong correlations, several other variables showed statistically significant but weaker positive correlations. Additional parameters such as age (*R* = 0.28, *P* < .001), BMI (*R* = 0.27, *P* = .004), fasting glucose (*R* = 0.31, *P* < .001), triglyceride levels (*R* = 0.39, *P* < .001), neutrophil count (*R* = 0.39, *P* < .001), monocyte count (*R* = 0.28, *P* = .006), and platelet count (*R* = 0.36, *P* = .014) also showed significant positive correlations with complications. In contrast, HDL-C levels (*r* = –0.32, *P* = .002) and lymphocyte count (*r* = –0.27, *P* < .001) were negatively correlated (Table 3).

**Table 3:** Correlation analysis between clinical and laboratory parameters and the presence of diabetic complications.

Parameters	*R*	*P* value
Age (yr)	0.28	<.001
Gender – female	−0.13	.029
Gender – male	0.21	.018
Diabetes duration (yr)	0.43	<.001
Body Mass Index (kg/m^2^)	0.27	.004
HbA1c (%)	0.36	<.001
Fasting glucose (mg/dL)	0.31	<.001
Triglycerides (mg/dL)	0.39	<.001
HDL-C (mg/dL)	−0.32	.002
TG/HDL-C ratio	0.62	<.001
TGI	0.68	<.001
PIV (median, IQR)	0.65	<.001
Neutrophils (10^3^/µL)	0.39	<.001
Lymphocytes (10^3^/µL)	−0.27	<.001
Monocytes (10^3^/µL)	0.28	.006
Platelets (10^3^/µL)	0.36	.014

HbA1c = glycated hemoglobin; TG/HDL-C = triglyceride-to-HDL cholesterol, TGI = triglyceride-glucose index, PIV = pan-immune-inflammation value; IQR = interquartile range.

Logistic regression analysis of predictors for diabetic complications was shown in Table 4. Among these, the TGI emerged as the strongest predictor, with an OR of 2.12 (95% CI: 1.65–2.71, *P* < .001). This was followed by the PIV, which also showed a robust association (OR: 1.94, 95% CI: 1.52–2.46, *P* < .001). The TG/HDL-C ratio was another significant predictor (OR: 1.83, 95% CI: 1.48–2.27, *P* < .001). Additionally, elevated HbA1c levels (OR: 1.62, 95% CI: 1.31–2.01, *P* < .001) and longer duration of diabetes (OR: 1.45, 95% CI: 1.22–1.74, *P* < .001) were independently associated with higher odds of developing complications (Table 4).

**Table 4:** Logistic regression analysis of predictors for diabetic complications.

Variables	OR	95% CI	*P* value
Diabetes duration (yr)	1.45	1.22–1.74	<.001
HbA1c (%)	1.62	1.31–2.01	<.001
TG/HDL-C ratio	1.83	1.48–2.27	<.001
TGI	2.12	1.65–2.71	<.001
PIV	1.94	1.52–2.46	<.001

CI = confidence interval, HbA1c = glycated hemoglobin, OR = odds ratio, PIV = pan-immune-inflammation value, TG/HDL-C = triglyceride-to-HDL cholesterol, TGI = triglyceride-glucose index.

**Figure 1.**
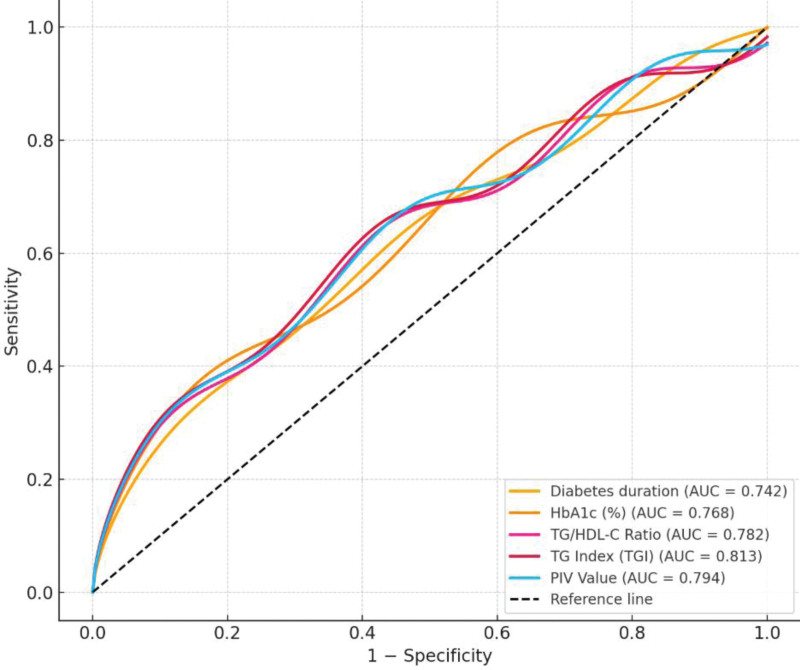
ROC curves of predictive parameters for diabetic complications.

ROC analysis of predictive parameters for diabetic complications was shown in Table 5. Among the evaluated parameters, the TGI demonstrated the highest diagnostic accuracy, with an AUC of 0.813 (95% CI: 0.767–0.859, *P* < .001), at a cutoff value of > 8.95, yielding a sensitivity of 81.4% and specificity of 77.6%. The PIV also showed strong discriminative ability, with an AUC of 0.794 (95% CI: 0.748–0.840, *P* < .001), sensitivity of 76.5%, and specificity of 71.4% at a cutoff of > 420. Similarly, the TG/HDL-C ratio provided a good predictive value (AUC = 0.782, 95% CI: 0.734–0.829, *P* < .001), with a sensitivity of 78.2% and specificity of 72.3% at a cutoff of > 4.5. HbA1c and diabetes duration, while traditionally used indicators, demonstrated slightly lower but still statistically significant predictive capacities (AUC = 0.768 and 0.742, respectively; both *P* < .001). The optimal cutoff values for HbA1c (>7.8%) and diabetes duration (>6.5 years) remained clinically relevant (Table 5, Fig. 1).

**Table 5:** ROC analysis of predictive parameters for diabetic complications.

Parameters	Cutoff	Sensitivity	Specificity	AUC (95% CI)	*P* value
Diabetes duration (yr)	>6.5 years	71.0%	68.2%	0.742 (0.695–0.789)	<.001
HbA1c (%)	>7.8%	75.6%	70.5%	0.768 (0.721–0.815)	<.001
TG/HDL-C ratio	>4.5	78.2%	72.3%	0.782 (0.734–0.829)	<.001
TGI	>8.95	81.4%	77.6%	0.813 (0.767–0.859)	<.001
PIV	>420	76.5%	71.4%	0.794 (0.748–0.840)	<.001

AUC = area under the curve, CI = confidence interval, HbA1c = glycated hemoglobin, PIV = pan-immune-inflammation value, TG/HDL-C = triglyceride-to-HDL cholesterol, TGI = triglyceride-glucose index.

In addition to individual index analyses, a combined predictive model incorporating TGI, TG/HDL-C, and PIV was evaluated. The combined model demonstrated the highest discriminatory performance, with an AUC of 0.846 (95% CI: 0.802–0.890), outperforming each index alone (TGI AUC = 0.813, PIV AUC = 0.794, TG/HDL-C AUC = 0.782). At the optimal cutoff, the combined model yielded a sensitivity of 84.2% and specificity of 78.5% (Table 6).

**Table 6:** Performance of the combined predictive model.

Parameter	Cutoff	Sensitivity	Specificity	AUC (95% CI)	*P* value
Combined model (TGI + TG/HDL-C + PIV)	>0.61 (predicted probability)	84.2%	78.5%	0.846 (0.802–0.890)	<.001

AUC = area under the curve, CI = confidence interval, PIV = pan-immune-inflammation value, TG/HDL-C = triglyceride-to-HDL cholesterol ratio, TGI = triglyceride-glucose index.

## 4. Discussion

In this retrospective cohort study, we investigated the predictive value of the TG/HDL-C, TGI, and PIV in relation to diabetic complications among patients with T2DM. Our results revealed that all 3 markers were significantly associated with the presence of diabetic complications and performed well in correlation, logistic regression, and ROC analyses. Among them, the TGI demonstrated the highest predictive performance, with an AUC of 0.813.

Our findings align with previous reports indicating that metabolic and inflammatory markers play an important role in the progression of diabetic complications. Babic et al reported that both TG/HDL-C and TGI were significantly correlated with poor glycemic control and complications in T2DM patients, highlighting their utility as practical, low-cost biomarkers in clinical settings.^[9]^ Similarly, Jabeen et al found a significant association between elevated TG/HDL-C and TGI values and both microvascular and macrovascular complications in diabetic patients.^[12]^

The TGI emerged as the most powerful individual predictor in our study, with the highest OR (2.12) and ROC performance. This is consistent with previous research by Bilgin et al., who demonstrated the significant prognostic value of TGI in patients with ST-elevation myocardial infarction and acute coronary syndrome, linking higher TGI levels with increased thrombus burden and mortality.^[8,10]^ In addition, Zhang et al reported that TGI was an independent predictor of major adverse cardiovascular events (MACE) in T2DM patients, with performance similar to coronary calcium scoring.^[18]^ Khan et al further emphasized that the TGI reflects insulin resistance with significant concordance to HOMA-IR, and could be used in diverse clinical populations.^[19]^ Furthermore, Liu et al identified a significant association between elevated TGI and diabetic nephropathy progression, supporting its relevance for microvascular risk stratification.^[20]^

TG/HDL-C, another metabolic indicator, also showed a significant relationship with complications (OR: 1.83; AUC: 0.782). This supports the findings of Lin et al., who highlighted TG/HDL-C as a simple yet informative surrogate marker for insulin resistance and cardiovascular risk in diabetic populations.^[21]^ Cheng et al demonstrated that TG/HDL-C ratio outperformed individual lipid markers in predicting insulin resistance and cardiovascular risk in Latin American diabetic cohorts.^[22]^ Karimi et al also showed a clear association between higher TG/HDL-C ratios and the presence of diabetic nephropathy and retinopathy, even after adjusting for glycemic control and duration of disease.^[23]^ Likewise, Kosmas et al linked TG/HDL-C to visceral adiposity and systemic inflammation, reinforcing its role at the intersection of metabolic and inflammatory pathways.^[24]^

Importantly, the PIV – a composite marker of systemic immune-inflammation – was also significantly associated with the presence of complications (OR: 1.94; AUC: 0.794). Although PIV has been primarily studied in oncology, recent work by Bilgin et al demonstrated its utility in predicting mortality in pulmonary thromboembolism and acute coronary syndromes.^[10,16]^ In our study, PIV provided a broader reflection of the chronic inflammatory state associated with diabetes. Sokmen et al demonstrated that the pan-immune-inflammation index (PIV) effectively distinguished patients with masked hypertension among individuals with T2DM, highlighting its potential role in identifying cardiometabolic risk in this population.^[25]^ Yan et al further confirmed that PIV could independently predict adverse cardiovascular events in patients with acute coronary syndrome, supporting its broader use in inflammatory cardiometabolic conditions.^[26]^ These findings, along with ours, suggest that PIV could serve as a valuable index for integrated immune-metabolic risk assessment in diabetic populations.

The combined use of TGI, TG/HDL-C, and PIV is particularly noteworthy. While most previous studies have examined these parameters independently, our study is among the few to evaluate them together, revealing their complementary predictive value. The integration of metabolic and inflammatory signals into a unified risk-assessment model enhances the early identification of high-risk individuals. Consistent with this concept, the combined model integrating TGI, TG/HDL-C, and PIV demonstrated superior predictive performance compared with each marker individually, with an AUC of 0.846. This improvement underscores the value of incorporating multiple pathophysiological domains into a single predictive framework and suggests that combined marker strategies may provide more refined and clinically meaningful stratification of high-risk patients.

From a clinical perspective, the use of TGI, TG/HDL-C, and PIV may offer a practical, low-cost approach to enhancing risk stratification during routine diabetes care. Elevated values in any of these indices – particularly when combined – could help clinicians identify patients at increased risk of developing microvascular or macrovascular complications. In such cases, clinicians may consider earlier referral to endocrinology or ophthalmology, more frequent monitoring of renal function or neurologic symptoms, or initiation of more aggressive cardiometabolic risk-reduction strategies. Because these indices are derived from standard laboratory parameters, they can be readily incorporated into existing clinical workflows without additional cost or testing burden. Integrating these markers into risk-assessment protocols may therefore facilitate earlier intervention and more personalized management for high-risk individuals with T2DM.

## 5. Limitations

Despite these promising findings, this study has some limitations. First, its retrospective design limits the ability to establish causality. Second, as it is a single-center study, generalizability may be limited. Third, we did not evaluate longitudinal outcomes such as mortality or progression of specific complications. Although complication subtypes were recorded, the sample size in some macrovascular categories was relatively small, which limited the ability to perform adequately powered subgroup analyses. Future studies with larger and more balanced patient cohorts should investigate whether TGI, TG/HDL-C, and PIV exhibit differential predictive performance for microvascular versus macrovascular complications. Furthermore, as complication status was extracted from electronic health records, variability in clinical documentation, diagnostic coding, and physician reporting may have influenced the classification of some outcomes. This inherent limitation of retrospective data collection may introduce misclassification bias and should be considered when interpreting the results.

## 6. Conclusions

In conclusion, TGI, TG/HDL-C ratio, and PIV are significantly associated with the presence of diabetic complications and demonstrate meaningful individual predictive value. Furthermore, the combined model integrating all 3 indices offers superior diagnostic performance (AUC = 0.846) compared with any single marker. These readily available indices may provide simple, accessible, and cost-effective tools to support early risk stratification in patients with T2DM. Future prospective and multicenter studies are warranted to validate these findings and to explore the clinical utility of incorporating combined marker strategies into routine diabetes management.

## Author contributions

**Conceptualization:** Yahya Urkmez.

**Data curation:** Yahya Urkmez, Mehmet Karacali, Esra Urkmez Kilinc.

**Formal analysis:** Yahya Urkmez, Mehmet Karacali, Esra Urkmez Kilinc.

**Funding acquisition:** Yahya Urkmez, Mehmet Karacali.

**Investigation:** Yahya Urkmez, Esra Urkmez Kilinc.

**Methodology:** Yahya Urkmez, Mehmet Karacali, Esra Urkmez Kilinc.

**Resources:** Yahya Urkmez, Mehmet Karacali, Esra Urkmez Kilinc.

**Software:** Yahya Urkmez, Esra Urkmez Kilinc.

**Supervision:** Esra Urkmez Kilinc.

**Validation:** Yahya Urkmez.

**Writing – original draft:** Yahya Urkmez.

**Writing – review & editing:** Mehmet Karacali, Esra Urkmez Kilinc.
